# Thyroid-metabolic interactions in pediatric epilepsy: insights from central sensitivity indices and peripheral hormone markers

**DOI:** 10.1007/s00431-026-07045-8

**Published:** 2026-05-08

**Authors:** Valeria Calcaterra, Lucia Labati, Ilaria Anna Maria Scavone, Barbara Scelsa, Pierangelo Veggiotti, Gianvincenzo Zuccotti

**Affiliations:** 1 Department of Internal Medicine and Therapeutics, University of Pavia, 27100 Pavia, Italy; 2 Pediatric Department, Buzzi Children’s Hospital, 20154 Milan, Italy; 3 Department of Biomedical and Clinical Science, University of Milano, 20157 Milan, Italy; 4 Pediatric Neurology Unit, V. Buzzi Children’s Hospital, 20154 Milan, Italy

**Keywords:** Epilepsy, Thyroid hormone metabolism, FT3/FT4 ratio, Thyroid hormone sensitivity, Pediatric population

## Abstract

Childhood epilepsy is often linked to endocrine and metabolic disturbances, partly from prolonged antiseizure treatment. While thyroid dysfunction is known in this population, routine tests may miss subtle changes in hormone sensitivity or peripheral metabolism. This study seeks to thoroughly assess central and peripheral thyroid hormone regulation in pediatric epilepsy and explore its connection to metabolic health. In this cross-sectional study, 152 children and adolescents with epilepsy and 116 age- and sex-matched controls underwent assessment of thyroid function (TSH, FT4, FT3), central thyroid hormone sensitivity indices (TFQI, PTFQI, TSHI, TT3RI, TT4RI), peripheral thyroid hormone metabolism (FT3/FT4 ratio), and metabolic parameters (fasting glucose, insulin, and lipid profile). Stratified and multivariable regression analyses were performed to identify independent associations, and correlation analyses were used to explore thyroid–metabolic relationships. Children with epilepsy had higher fasting insulin and a worse lipid profile than controls. Thyroid function markers (TSH, FT4) and central thyroid sensitivity indices were similar between groups, but FT3 and the FT3/FT4 ratio were elevated, suggesting altered peripheral thyroid metabolism despite preserved central regulation. Multivariable analyses identified BMI z-score as a major independent determinant of metabolic alterations and thyroid hormone sensitivity, while antiepileptic drug exposure and disease duration showed variable and modest associations. *Conclusions*: Pediatric epilepsy is characterized by preserved central thyroid regulation but altered peripheral thyroid hormone metabolism, as reflected by an increased FT3/FT4 ratio. These findings suggest a multifactorial and potentially adaptive endocrine–metabolic remodeling influenced by adiposity and treatment exposure, warranting further investigation in longitudinal studies.

**Table:** 

**What is Known:** • *Pediatric epilepsy and antiseizure medications are associated with subtle thyroid alterations and increased metaboli crisk, commonly assessed using TSH and FT4.*
**What is New:** • *Children with epilepsy show increased FT3 and FT3/FT4 ratio despite preserved central thyroid regulation, suggesting altered peripheral thyroid metabolism.* • *BMI z-score emerged as a major independent determinant of thyroid sensitivity indices and metabolic impairment.*

**Supplementary Information:**

The online version contains supplementary material available at 10.1007/s00431-026-07045-8.

## Introduction

Epilepsy is one of the most common chronic neurological disorders in childhood, affecting about 0.5–1% of children worldwide. It is characterized by recurrent, unprovoked seizures caused by abnormal synchronous neuronal activity in the brain, with incidence and prevalence varying across populations and regions [1, 2].

Antiseizure medications (ASMs) represent the first-line and, when necessary, subsequent-line therapeutic approach for the management of pediatric epilepsy [3]. Beyond seizure control, epilepsy and its treatment may influence growth, pubertal development, endocrine function, and metabolic homeostasis during critical phases of neuroendocrine maturation [4, 5].

Thyroid hormones (THs) are essential for brain development, growth, and metabolic regulation, and even subtle alterations may have clinically relevant effects in children [6, 7]. Thyroid function abnormalities have been reported in epilepsy, especially in patients receiving long-term ASM therapy, although findings remain inconsistent [8–10].

Enzyme-inducing ASMs are often associated with reduced thyroxine (T4) levels, usually with normal or only mildly increased thyroid-stimulating hormone (TSH), while non-enzyme-inducing drugs such as valproate have also been linked to subclinical thyroid dysfunction, especially in children [8, 10–13]. Newer ASMs seem to have milder effects, and most thyroid alterations reported in epilepsy remain subclinical and within reference ranges [10, 14].

Traditional thyroid assessment based on TSH and FT4 mainly reflects central HPT-axis regulation and may not detect alterations in peripheral thyroid hormone metabolism [15, 16]. Since triiodothyronine (T3) is largely produced by peripheral conversion of T4, the FT3/FT4 ratio has been proposed as an indirect marker of this process, and its changes may reflect adaptive metabolic responses [17, 18].

Children and adolescents with epilepsy are at increased risk of metabolic disturbances, including insulin resistance and dyslipidemia [19, 20]. Given the central role of THs in glucose and lipid metabolism, subtle changes in peripheral thyroid hormone availability may contribute to these metabolic alterations [21, 22].

Beyond conventional thyroid tests, several composite indices, such as the thyroid feedback quantile-based index (TFQI), parametric TFQI (PTFQI), TSH index (TSHI), and thyrotroph T4 resistance index (TT4RI), have been developed to assess central thyroid hormone sensitivity or resistance [23]. Alterations in these indices have been associated with adverse cardiometabolic profiles even in euthyroid individuals [24, 25]. However, data on thyroid hormone sensitivity indices and peripheral conversion markers in pediatric epilepsy are scarce.

Therefore, the present study aimed to comprehensively explore thyroid function in children and adolescents with epilepsy by integrating conventional thyroid parameters, indices of central thyroid hormone sensitivity, and markers of peripheral thyroid hormone metabolism, including the FT3/FT4 ratio. In addition, we assessed the relationship between thyroid-related parameters and metabolic markers using correlation analyses to better characterize the thyroid–metabolic interplay in pediatric epilepsy.

## Patients and methods

This was a cross-sectional, observational study conducted at a tertiary pediatric center. Children and adolescents with a diagnosis of epilepsy were consecutively recruited during routine outpatient follow-up visits. A group of age- and sex-matched healthy subjects was enrolled as controls.

The epilepsy group included patients aged ≤ 18 years with a confirmed diagnosis of epilepsy [26] and receiving stable AED therapy for at least 6 months prior to enrollment. Monotherapy was used in 32 patients (21%), while polytherapy was prescribed in 120 (79%). Overall, valproic acid was the most frequently prescribed AED (*n* = 65), followed by levetiracetam (*n* = 26), phenobarbital (*n* = 18), topiramate (*n* = 8), and lamotrigine (*n* = 8). Other AEDs were used in the remaining patients.

The control group consisted of healthy children without a history of neurological, endocrine, metabolic, or chronic inflammatory diseases, recruited during routine health evaluations.

Exclusion criteria for both groups included known thyroid disease, thyroid hormone therapy, systemic inflammatory conditions, and use of medications affecting thyroid function other than AEDs.

The study was approved by our Institute’s ethics committee (Protocol n. 2021/ST/207; register n. 0016834, 04/04/2022, CE Area 1 Milan, Italy) and conducted in accordance with the 2013 revision of the Declaration of Helsinki. Written informed consent was obtained from parents or legal guardians, and assent from minors when appropriate.

### Clinical and anthropometric assessment

For all participants, demographic data (age and sex) and clinical history were collected. 

Anthropometric measurements included body weight and height, measured using standardized procedures [27]. Body mass index (BMI) was calculated as weight (kg) divided by height squared (m^2^), and BMI z-scores were derived according to age- and sex-specific reference standards. Pubertal status was assessed according to Tanner [28, 29] staging by a trained pediatric endocrinologist.

### Laboratory measurements

After an overnight fast, venous blood samples were collected between 08:00 and 09:00 a.m. Serum glucose, insulin, total cholesterol, HDL-cholesterol, and triglycerides were measured using standard automated laboratory methods. Fasting blood glucose and lipid parameters were expressed in mg/dL, while insulin levels were expressed in μU/mL.

Thyroid function assessment included serum thyroid-stimulating hormone (TSH), free thyroxine (FT4), and free triiodothyronine (FT3), measured using chemiluminescent immunoassays [30]. All measurements were performed in the same laboratory using standardized methods.

### Calculation of thyroid hormone sensitivity parameters

Peripheral sensitivity to THs was assessed using the FT3/FT4 ratio [31, 32].

Central sensitivity to TH was evaluated through several indices, including the TSH index (TSHI), thyrotroph T4 resistance index (TT4RI), thyrotroph T3 resistance index (TT3RI), Thyroid Feedback Quantile-based Index (TFQI), and its parametric form (PTFQI), calculated as described below.

- TSHI was calculated as: ln(TSH, mIU/L) + 0.1345 × FT4 (pmol/L). Elevated TSHI values reflect reduced sensitivity to THs, whereas lower values indicate enhanced sensitivity [33, 34].
- TT4RI was defined as the product of FT4 (pmol/L) and TSH (mIU/L).
- TT3RI was calculated as FT3 (pmol/L) multiplied by TSH (mIU/L).
- For both TT4RI and TT3RI, higher values denote diminished central responsiveness to THs, in a manner comparable to TSHI [33].
- TFQI was computed using the cumulative distribution function (cdf). TFQI = cdf (FT4) − (1 − cdf​(TSH)) [32, 34, 35]. This index provides a continuous evaluation of the hypothalamic–pituitary–thyroid (HPT) axis response to circulating THs levels, quantifying deviations from the expected inhibitory effect of TH on TSH secretion. TFQI values range from − 1 to 1, where positive values indicate decreased sensitivity, negative values increased sensitivity, and values near zero represent normal thyroid hormone sensitivity.
- PTFQI was calculated using population-based mean and standard deviation values for FT4 and TSH, using the formula: Φ[(FT4 − μFT4)/σFT4] − (1 − Φ[(TSH − μTSH)/σTSH]). The PTFQI represents a parametric approximation of TFQI, allowing its application across different populations, and it shares the same interpretative framework as TFQI [34].

## Statistical analysis

Statistical analyses were performed in patients with epilepsy and healthy controls. Continuous variables were assessed for normality using the Shapiro–Wilk test and inspection of distribution plots and are presented as mean ± SD or median (IQR), as appropriate, while categorical variables are reported as frequencies and percentages. Between-group comparisons were conducted using standard parametric or non-parametric tests (Student’s *t* test or the Mann–Whitney *U* test for continuous variables and the χ^2^ test or Fisher’s exact test for categorical variables). Outliers were identified using the IQR method (1.5 × IQR) and, as a sensitivity analysis, results were confirmed using an alternative ± 3 SD criterion/including all values.

The primary analytical approach relied on multivariable regression models to identify independent associations between demographic, clinical, and metabolic variables (sex, age, BMI z-score, Tanner stage, AED type and duration, fasting glucose, triglycerides, and cholesterol levels) and thyroid function parameters. Results are reported as adjusted coefficients or odds ratios (ORs) with 95% confidence intervals (CIs). Thyroid parameters were dichotomized prior to analysis. FT3, FT4, and TSH were classified as normal or altered according to reference ranges (FT3: 3.5–6.3 pmol/L; FT4: 9–19.3 pmol/L; TSH: 0.5–4.2 μIU/mL), while composite thyroid hormone sensitivity indices (TFQI, PTFQI, TT3RI, TT4RI, and TSHI) were considered altered when values exceeded the third quartile of the study population distribution.

PTFQI was calculated using the mean and standard deviation values of TSH and FT4 and these values were applied to standardize individual FT4 and TSH measurements prior to transformation using the cumulative normal distribution, as originally described in the PTFQI methodology.

Logistic regression models were additionally used to explore the association between epilepsy-related variables (disease duration and time since AED initiation) and thyroid parameter alterations.

Correlation analyses and stratified comparisons (by sex, BMI, and AED exposure) were performed as secondary exploratory analyses to support the interpretation of multivariable findings. Comparisons between two subgroups were conducted using Student’s *t* test or Mann–Whitney *U* test, while comparisons among multiple subgroups were performed using one-way ANOVA or Kruskal–Wallis test, with Bonferroni correction applied when appropriate.

Missing data were handled using complete-case analysis or median imputation in regression models when appropriate. Statistical significance was set at *p* < 0.05.

Statistical analyses were primarily conducted using Microsoft Excel with the Real Statistics Resource Pack add-in, chosen for its transparency, accessibility, and ability to perform descriptive statistics, hypothesis testing, correlation analyses, logistic regression, and graphical visualization in a unified framework. Key analyses were independently verified using R (version 4.5.2) to ensure robustness and reproducibility.

## Results

### Demographics and anthropometric parameters

A total of 268 participants were included in the analysis, comprising 152 patients with epilepsy and 116 healthy controls. No significant difference in age was observed between patients with epilepsy and controls (*p* = 0.184). Sex distribution was comparable between groups (*p* = 0.587).

Patients with epilepsy exhibited significantly lower body weight (*p* < 0.001) and height (*p* < 0.001) compared with controls. In contrast, BMI and BMI z-score did not differ significantly between groups (*p* = 0.982 and *p* = 0.256, respectively).

Pubertal stage distribution differed significantly between groups (*p* < 0.001), with higher prevalence of prepubertal status (stage 0) in patients with epilepsy compared to controls.

Demographics and anthropometric parameters are resumed in Table 1.

**Table 1:** Descriptive statistics. Data are presented as mean ± standard deviation for normally distributed variables and as median (interquartile range) for non-normally distributed variables. Categorical variables are reported as *n* (%)

	Epilepsy (***n*** = 152)	Controls (***n*** = 116)	***p*** value	Total population (***n*** = 268)
Age (years)	9.22 ± 5.32	9.93 ± 3.73	0.184	9.52 ± 4.70
Sex				
M	84 (55.26%)	61 (52.59%)	0.587	145 (54.10%)
F	68 (44.74%)	55 (47.41%)		123 (45.90%)
Pubertal stage				
0	76 (50.00%)	43 (37.07%	< 0.001	119 (44.40%)
Stage 1	31 (20.39%)	454 (46.55%)		85 (31.72%)
Stage 2	45 (29.61%)	19 (16.38%)		64 (23.88%)
Weight (kg)	27.35 ± 15.85	35.64 ± 15.35	< 0.001	30.98 ± 16.14
Height (m)	1.24 ± 0.28	1.40 ± 0.23	< 0.001	1.32 ± 0.27
BMI (Kg/m^2^)	16.70 (5.10)	16.21 (4.77)	0.982	16.48 (4.87)
BMI z-score	− 0.29 (2.82)	− 0.25 (1.58)	0.256	− 0.29 (2.13)
Fasting blood glucose (mg/dl)	86.00 (19.00)	78.00 (16.25)	0.850	83.00 (21.00)
Insulin (U/mL)	13.70 (20.00)	4.30 (5.95)	< 0.001	7.50 (11.00)
HDL-Cholesterol (mg/dl)	45.79 ± 19.40	53.86 ± 12.43	< 0.001	49.65 ± 16.89
Total cholesterol (mg/dl)	151.71 ± 41.92	137.43 ± 26.14	0.006	144.92 ± 35.93
Triglycerides (mg/dl)	88.50 (53.50)	64.00 (26.00)	< 0.001	71.00 (43.00)
TSH (mIU/L)*	1.88 (1.39)	2.10 (1.62)	0.718	1.93 (1.62)
	2.33 ± 2.14	2.41 ± 1.43		2.37 ± 1.87
TyG	8.20 (0.74)	7.77 (0.26)	< 0.001	7.87 (0.60)
HOMA-IR	2.60 (3.73)	0.77 (0.92)	< 0.001	1.33 (2.14)
FT3 (pmol/L)	4.74 ± 1.03	4.31 ± 0.78	< 0.001	4.55 ± 0.95
FT4 (pmol/L)	12.14 ± 2.21	12.37 ± 2.07	0.433	12.25 ± 2.14
TFQI	− 0.03 ± 0.30	− 0.03 ± 0.31	0.955	− 0.03 ± 0.30
PTFQI	0.02 ± 0.33	0.01 ± 0.33	0.872	0.01 ± 0.33
FT3/FT4	0.41 ± 0.21	0.36 ± 0.14	< 0.001	0.38 ± 0.19
TSHI	2.31 (0.78)	2.47 (0.66)	0.133	2.43 (0.77)
TT3RI	8.65 (8.70)	7.84 (8.22)	0.297	8.50 (8.20)
TT4RI	23.38 (17.54)	27.90 (17.27)	0.838	25.09 (18.88)

*TSH is a non-normally distributed variable; however, the mean ± SD was also reported because these values were used to calculate the PTFQI

### Metabolic profile

Fasting blood glucose levels were similar between groups (*p* = 0.850). However, fasting insulin concentrations were significantly higher in patients with epilepsy compared with controls (*p* < 0.001). Consistently, insulin resistance indices were significantly increased in patients with epilepsy, as shown by higher TyG index values (*p* < 0.001) and HOMA-IR levels (*p* < 0.001).

Regarding lipid profile, patients with epilepsy had significantly lower HDL-cholesterol levels (*p* < 0.001), as well as higher total cholesterol (*p* = 0.006) and triglyceride levels (*p* < 0.001) compared with controls.

Metabolic profiling results are presented in Table 1.

### Thyroid function and indices of thyroid hormone sensitivity

As reported in Table 1, serum TSH and FT4 concentrations did not differ between patients with epilepsy and controls (*p* = 0.718 and *p* = 0.433), whereas FT3 levels were significantly higher in patients with epilepsy (*p* < 0.001). Indices of central thyroid hormone sensitivity (TFQI, PTFQI, TSHI, TT3RI, TT4RI) were also comparable between groups.

In contrast, the FT3/FT4 ratio was significantly higher in patients with epilepsy (*p* < 0.001), indicating altered peripheral thyroid hormone metabolism despite preserved central regulation.

Distributional analyses (Supplementary material Fig. 1) showed greater variability in patients with epilepsy across thyroid parameters and resistance indices (TSHI, TT3RI, TT4RI), with broader distributions and occasional shifts in central tendency compared with controls.

Both TFQI and PTFQI exhibited broader distributions among patients with epilepsy, although their central values remained comparable to those of controls, potentially reflecting greater variability in central thyroid hormone feedback sensitivity. Similarly, the FT3/FT4 ratio was shifted toward higher values in patients with epilepsy, suggesting differences in peripheral thyroid hormone metabolism.

### Correlation analysis

Correlation matrix analyses performed in the epilepsy group, controls, and the overall population did not show strong or consistent associations between thyroid-related parameters and metabolic markers (Supplementary material Fig. 2).

In patients with epilepsy, correlations between FT3, the FT3/FT4 ratio, TSH, and metabolic variables were generally weak, with no clear thyroid–metabolic clustering. Associations were mainly confined within the same physiological domains (thyroid or metabolic).

In controls, correlation patterns appeared more physiological, with stronger within-domain associations and minimal cross-domain relationships.

Overall, these findings indicate that alterations in thyroid hormone metabolism in pediatric epilepsy are not linearly associated with metabolic markers in this cross-sectional setting.

### Associations between thyroid-related parameters and epilepsy status

Forest plot analysis (Table 2) showed no significant association between conventional thyroid parameters and epilepsy status. TSH (OR 1.51; 95% CI 0.67–3.39) and FT4 (OR 1.35; 95% CI 0.39–4.73) displayed nonsignificant trends toward higher odds, while FT3 showed a nonsignificant inverse trend (OR 0.86; 95% CI 0.44–1.68).

**Table 2:**
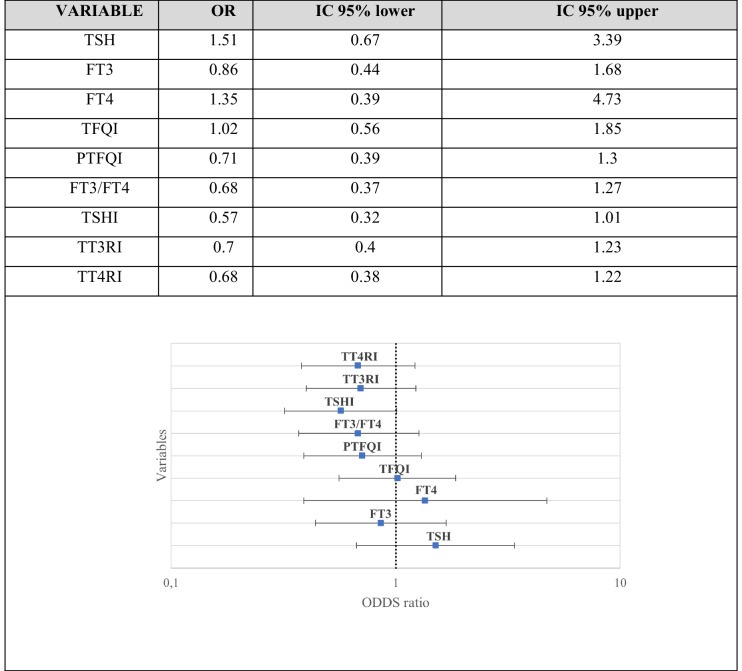
Forest plot–derived odds ratios for thyroid-related parameters in patients with epilepsy and controls

*TSH* thyroid-stimulating hormone, *FT3* free triiodothyronine, *FT4* free thyroxine, *TFQI* thyroid feedback quantile-based index, *PTFQI* parametric thyroid feedback quantile-based index, *FT3*/*FT4* free triiodothyronine to free thyroxine ratio, *TSHI* thyroid-stimulating hormone index, *TT3RI* thyrotroph triiodothyronine resistance index, *TT4RI* thyrotroph thyroxine resistance index

Similarly, thyroid hormone sensitivity indices were not significantly associated with epilepsy. TFQI was neutral (OR 1.02; 95% CI 0.56–1.85), whereas PTFQI, TSHI, TT3RI, and TT4RI showed ORs below unity (0.57–0.71), and the FT3/FT4 ratio showed an OR of 0.68 (95% CI 0.37–1.27).

All confidence intervals included unity, indicating the absence of significant independent associations.

### Logistic regression analysis

Logistic regression analyses (Table 3) showed consistent but nonsignificant trends toward increased odds of thyroid parameter alterations with both disease duration and AED exposure.

**Table 3:** Logistic regression results

Variable	Both time	Time diagnosis	Starting time therapy
	OR (95%CI)	OR (95%CI)	OR (95%CI)
TSH	OR D = 1.12 (0.83; 1.50) OR T = 0.89 (0.66; 1.19)	1.00 (0.89; 1.12)	0.97 (0.87; 1.09)
FT3	OR D = 1.35 (0.88; 2.07) OR T = 0.74 (0.49; 1.14)	1.02 (0.92; 1.15)	0.98 (0.88; 1.10)
FT4	OR D = 0.90 (0.73; 1.11) OR T = 1.21 (0.99; 1.48)	1.07 (0.97; 1.20)	1.11 (1.00; 1.23)
TFQI	OR D = 1.20 (0.89; 1.61) OR T = 0.90 (0.67; 1.22)	1.09 (0.99; 1.21)	1.07 (0.97; 1.18)
PTFQI	OR D = 1.20 (0.89; 1.61) OR T = 0.90 (0.67; 1.21)	1.09 (0.99; 1.20)	1.06 (0.96; 1.17)
FT3/FT4	OR D = 0.95 (0.73; 1.24) OR T = 0.96 (0.76; 1.22)	0.92 (0.80; 1.06)	0.93 (0.82; 1.05)
TSHI	OR D = 1.12 (0.85; 1.47) OR T = 0.92 (0.70; 1.20)	1.04 (0.94; 1.15)	1.02 (0.92; 1.13)
TT3RI	OR D = 1.17 (0.90; 1.51) OR T = 0.89 (0.69; 1.15)	1.05 (0.95; 1.16)	1.02 (0.92;1.12)
TT4RI	OR D = 1.16 (0.86; 1.56) OR T = 0.88 (0.66; 1.19)	1.03 (0.93; 1.14)	1.01 (0.91; 1.11)

*TSH* thyroid-stimulating hormone, *FT3* free triiodothyronine, *FT4* free thyroxine, *TFQI* thyroid feedback quantile-based index, *PTFQI* parametric thyroid feedback quantile-based index, *FT3*/*FT4* free triiodothyronine to free thyroxine ratio, *OR D* time diagnosis, *OR T* starting time therapy, *TSHI* thyroid-stimulating hormone index, *TT3RI* thyrotroph triiodothyronine resistance index, *TT4RI* thyrotroph thyroxine resistance index

Time since diagnosis was associated with higher odds for FT4 (OR 1.07; 95% CI 0.97–1.20), TFQI (OR 1.09; 95% CI 0.99–1.21), PTFQI (OR 1.09; 95% CI 0.99–1.20), and TT3RI (OR 1.05; 95% CI 0.95–1.16). Similarly, time since AED initiation showed comparable trends for FT4 (OR 1.11; 95% CI 1.00–1.23), TFQI (OR 1.07; 95% CI 0.97–1.18), and PTFQI (OR 1.06; 95% CI 0.96–1.17).

Although confidence intervals generally included unity, the consistency in effect direction suggests modest time-dependent influences on thyroid hormone homeostasis.

### Stratified and multivariate regression results

In stratified analyses (Tables 4 and 5), thyroid parameters varied according to sex, BMI z-score, and AED exposure. Female subjects showed higher HOMA-IR and lower PTFQI (*p* < 0.001), while no sex differences were observed for FT3 and FT4. Increasing BMI z-score was associated with higher TSH, TyG, HOMA-IR, and thyroid resistance indices (TSHI, TT3RI, TT4RI; all *p* < 0.001), along with a modest increase in the FT3/FT4 ratio (*p* = 0.035). Differences were also observed across AED groups (all *p* < 0.001), particularly with valproate.

**Table 4:** Descriptive statistics of thyroid parameters through sample stratification by sex, BMI z-score, and monotherapy versus polypharmacological treatment

Parameters	Sex			Type of AED			BMI z-score				
	Females (***n*** = 68)	Males (***n*** = 84)	p	One drug (***n*** = 32)	Poly-therapy (***n*** = 120)	***p***	≤ − 2 (***n*** = 27)	− 1 ≤ BMI z-score ≤ 1 (***n*** = 59)	1 ≤ BMI z-score ≤ 2 (***n*** = 19)	≥ 2 (***n*** = 15)	***p***
TSH (mIU/L)	1.57 (1.25)	1.94 (1.68)	0.108	1.80 (1.43)	2.09 (0.98)	0.729	1.79 (1.84)	1.91 (1.17)	1.63 (1.50)	2.43 (1.22)	< 0.001
TyG	8.14 (0.71)	8.22 (0.81)	0.519	8.21 (0.73)	7.79 (0.73)	0.593	8.36 (0.76)	7.84 (0.61)	8.82 (1.26)	8.44 (0.83)	< 0.001
HOMA-IR	3.74 (4.76)	2.21 (3.37)	< 0.001	2.76 (4.22)	2.55 (0.56)	0.081	1.87 (2.37)	3.01 (4.04)	6.82 (6.17)	5.25 (2.29)	< 0.001
FT3 (pmol/L)	4.73 ± 0.92	4.74 ± 1.11	0.977	4.67 ± 1.02	5.06 ± 1.05	0.124	4.50 ± 0.75	4.83 ± 1.13	4.73 ± 0.94	5.38 ± 1.04	0.116
FT4 (pmol/L)	12.21 ± 2.02	12.08 ± 2.35	0.755	12.14 ± 2.18	12.13 ± 2.33	0.986	12.17 ± 2.13	12.00 ± 1.97	12.85 ± 2.81	11.87 ± 2.48	0.600
TFQI	− 0.07 ± 0.31	0.00 ± 0.29	0.325	− 0.04 ± 0.29	0.00 ± 0.32	0.685	− 0.08 ± 0.25	− 0.02 ± 0.27	0.01 ± 0.30	0.02 ± 0.37	0.815
PTFQI	− 0.04 ± 0.35	0.06 ± 0.32	< 0.001	0.00 ± 0.32	0.07 ± 0.36	0.482	− 0.03 ± 0.31	0.03 ± 0.29	0.04 ± 0.36	0.08 ± 0.34	0.829
FT3/FT4	0.40 ± 0.21	0.41 ± 0.22	0.489	0.40 ± 0.21	0.43 ± 0.23	0.301	0.39 ± 0.19	0.42 ± 0.22	0.38 ± 0.18	0.50 ± 0.29	0.035
TSHI	2.27 (1.01)	2.38 (0.81)	< 0.001	2.30 (0.77)	2.46 (0.81)	0.908	2.50 (0.63)	2.37 (0.83)	2.36 (0.67)	2.44 (0.77)	< 0.001
TT3RI	7.89 (5.91)	8.82 (9.17)	0.291	8.82 (9.18)	8.59 (6.30)	0.301	8.22 (8.20)	8.85 (7.80)	6.96 (9.04)	13.98 (6.55)	< 0.001
TT4RI	21.70 (17.86)	24.19 (18.73)	0.720	23.33 (17.79)	24.09 (15.28)	0.482	25.35 (14.25)	24.45 (19.07)	23.86 (18.06)	24.46 (14.44)	< 0.001

*TSH* thyroid-stimulating hormone, *TyG* triglyceride–glucose index, *HOMA*-*IR* homeostasis model assessment of insulin resistance, *FT3* free triiodothyronine, *FT4* free thyroxine, *TFQI* thyroid feedback quantile-based index, *PTFQI* parametric thyroid feedback quantile-based index, *FT3*/*FT4* free triiodothyronine to free thyroxine ratio, *TSHI* thyroid-stimulating hormone index, *TT3RI* thyrotroph triiodothyronine resistance index, *TT4RI* thyrotroph thyroxine resistance index

**Table 5:** Descriptive statistics of thyroid parameters through sample stratification by type of main pharmacological treatment

	Valproic (***n*** = 65)	Levetiracetam (***n*** = 26)	Phenobarbital (***n*** = 18)	Topiramate (***n*** = 8)	Lamotrigine (***n*** = 8)	Other drugs (***n*** = 27)	***p*** value
TSH (mIU/L)	2.15 (1.85)	1.30 (1.30)	1.78 (0.90)	2.24 (2.76)	1.54 (1.06)	1.47 (1.04)	< 0.001
TyG	8.22 (0.72)	8.41 (0.64)	8.04 (1.04)	8.38 (0.59)	8.58 (0.52)	7.82 (0.72)	< 0.001
HOMA-IR	2.26 (2.76)	1.90 (5.31)	3.38 (3.65)	3.35 (5.04)	0.40 (0.00)	2.50 (3.19)	< 0.001
FT3 (pmol/L)	4.82 ± 1.07	4.78 ± 0.96	4.42 ± 0.56	4.86 ± 0.84	4.65 ± 1.46	4.73 ± 1.24	0.654
FT4 (pmol/L)	12.02 ± 1.86	12.59 ± 2.07	13.94 ± 3.92	12.46 ± 2.27	12.25 ± 2.02	11.40 ± 1.86	0.429
TFQI	0.00 ± 0.32	− 0.10 ± 0.26	0.12 ± 0.22	0.04 ± 0.22	− 0.05 ± 0.38	− 0.24 ± 0.28	0.668
PTFQI	0.07 ± 0.33	− 0.09 ± 0.33	0.16 ± 0.26	0.01 ± 0.26	− 0.03 ± 0.38	− 0.20 ± 0.32	0.474
FT3/FT4	0.42 ± 0.20	0.39 ± 0.19	0.32 ± 0.16	0.42 ± 0.23	0.39 ± 0.21	0.44 ± 0.23	0.404
TSHI	2.48 (0.62)	2.03 (1.10)	2.85 (1.29)	2.59 (1.39)	2.17 (0.36)	1.97 (0.61)	< 0.001
TT3RI	11.08 (9.47)	7.87 (8.20)	7.97 (3.80)	6.96 (9.89)	7.37 (3.57)	7.76 (4.70)	< 0.001
TT4RI	27.13 (19.78)	17.16 (21.40)	34.93 (51.46)	24.46 (46.88)	21.08 (6.81)	18.82 (10.71)	< 0.001

*TSH* thyroid-stimulating hormone, *TyG* triglyceride–glucose index, *HOMA*-*IR* homeostasis model assessment of insulin resistance, *FT3* free triiodothyronine, *FT4* free thyroxine, *TFQI* thyroid feedback quantile-based index, *PTFQI* parametric thyroid feedback quantile-based index, *FT3*/*FT4* free triiodothyronine to free thyroxine ratio, *TSHI* thyroid-stimulating hormone index, *TT3RI* thyrotroph triiodothyronine resistance index, *TT4RI* thyrotroph thyroxine resistance index

However, in multivariable regression analyses (Supplementary material Fig. 3), BMI z-score emerged as the strongest independent predictor of thyroid-related outcomes, showing robust associations with TSH (β ≈ 0.4–0.6, *p* < 0.01), TyG (β ≈ 0.05–0.08, *p* < 0.001), and thyroid hormone resistance indices (TSHI, TT3RI, TT4RI; all *p* < 0.01). Tanner stage and lipid parameters (triglycerides and HDL cholesterol) also retained independent associations, whereas sex and age had limited adjusted effects.

AED exposure and treatment duration showed weaker and less consistent associations, mainly affecting thyroid sensitivity indices rather than FT3 and FT4 levels.

## Discussion

Overall, this exploratory study suggests that pediatric epilepsy is associated with distinct anthropometric, metabolic, and thyroid-related features compared with healthy peers. Although central HPT-axis regulation appears preserved, patients showed alterations in peripheral thyroid hormone metabolism alongside an unfavorable metabolic profile, including hyperinsulinemia and dyslipidemia. These findings indicate that epilepsy and/or its treatment may affect endocrine–metabolic homeostasis beyond seizure control, with possible long-term cardiometabolic implications. Multivariable analyses identified BMI z-score as a major independent determinant of thyroid-related outcomes, while antiseizure medication exposure showed variable effects, supporting a multifactorial influence on thyroid–metabolic regulation.

The dissociation between preserved central thyroid regulation and altered peripheral thyroid hormone metabolism suggests that epilepsy-related factors, including antiepileptic drug exposure, may affect thyroid function even without overt dysfunction [36, 37]. Thyroid hormones play a key role in energy expenditure, lipid and glucose metabolism, and growth, and several antiepileptic drugs are known to interfere with TH synthesis, transport, deiodination, or clearance [38, 39]. Even in the absence of overt thyroid dysfunction, subtle alterations in peripheral thyroid hormone handling may therefore occur, particularly during critical developmental periods such as childhood and adolescence. Consistently, stratified analyses revealed significant differences across main AED groups in TSH, TSHI, TT3RI, and TT4RI, whereas FT3, FT4, TFQI, PTFQI, and the FT3/FT4 ratio did not differ significantly. The most pronounced differences were observed in patients receiving valproate-based therapy, suggesting [40, 41]. However, these findings should be interpreted cautiously, given the limited size of several treatment subgroups and the high prevalence of polytherapy.

Consistent with previous reports, patients with epilepsy showed lower weight and height and a different distribution of pubertal stages than controls. These findings are in line with the concept of delayed growth and pubertal development in pediatric epilepsy, potentially related to chronic disease burden, altered energy balance, or prolonged exposure to antiepileptic medications [42]. Importantly, BMI and BMI z-score did not differ between groups, suggesting proportionate growth impairment rather than overt undernutrition or overweight.

From a metabolic perspective, children and adolescents with epilepsy showed hyperinsulinemia despite normal fasting glucose, suggesting early insulin resistance, together with an unfavorable lipid profile [20, 43]. These findings indicate a subclinical metabolic alteration with possible long-term cardiometabolic implications, further supported by multivariable analyses identifying BMI z-score, TyG index, and HOMA-IR as independent contributors.

Importantly, central thyroid parameters, including serum TSH and FT4 concentrations and indices of central thyroid hormone sensitivity (TFQI, PTFQI, TSHI, TT3RI, and TT4RI), were comparable between patients with epilepsy and controls, supporting preserved HPT axis function. In contrast, FT3 levels and, more notably, the FT3/FT4 ratio were significantly higher in the epilepsy group. As the FT3/FT4 ratio is widely regarded as an indirect marker of peripheral deiodinase activity [44], this finding suggests a shift toward enhanced peripheral conversion of thyroxine (T4) to triiodothyronine (T3), rather than altered thyroidal secretion or pituitary feedback. Notably, this pattern was paralleled by a modest but significant increase in the FT3/FT4 ratio across BMI z-score categories, indicating that peripheral thyroid hormone conversion may be progressively modulated by adiposity rather than by central hypothalamic–pituitary feedback mechanisms [39, 45].

Forest plot analysis showed no independent association between epilepsy status and conventional thyroid parameters or thyroid hormone sensitivity indices when these were examined as dichotomized variables. Although some trends emerged, all confidence intervals crossed unity, indicating no statistical significance. This suggests that thyroid-related changes in pediatric epilepsy reflect subtle shifts in distribution rather than a clear increase in abnormal clinical values.

Correlation matrix analyses found no strong or consistent linear links between thyroid-related parameters, including the FT3/FT4 ratio, and metabolic markers in the epilepsy group. No clear thyroid–metabolic clusters emerged, suggesting that changes in peripheral thyroid hormone metabolism are likely influenced by indirect, non-linear, or multifactorial mechanisms. In controls, correlations appeared more physiological and were mostly confined within thyroid or metabolic domains, while correlations in the overall sample likely reflected greater statistical power rather than epilepsy-specific relationships.

Taken together, these findings indicate that pediatric epilepsy is characterized by a dissociation between preserved central thyroid regulation and altered peripheral thyroid hormone metabolism, occurring alongside metabolic abnormalities but without a straightforward linear relationship between the two. Whether the observed increase in peripheral T3 availability represents an adaptive mechanism aimed at maintaining energy homeostasis [46] or a maladaptive process potentially contributing to metabolic vulnerability remains to be clarified. In line with this interpretation, BMI z-score consistently ranked among the strongest predictors of indices reflecting thyroid hormone resistance and pituitary set-point regulation in multivariable analyses, reinforcing the central role of body composition in shaping thyroid hormone sensitivity in pediatric epilepsy [47].

In this context, the modest time-related trends observed suggest that thyroid hormone remodeling in pediatric epilepsy may develop gradually over the course of the disease and its treatment [48]. Although not statistically significant, the consistent direction of these associations points to a possible adaptive modulation of thyroid homeostasis, with antiepileptic drug exposure and treatment duration potentially exerting cumulative long-term effects.

The strengths of this study include the comprehensive assessment of central and peripheral thyroid hormone sensitivity and the use of correlogram analyses to investigate complex endocrine–metabolic interactions. Nevertheless, several limitations should be acknowledged. First, the cross-sectional design precludes any causal inference regarding the observed associations. Second, although stratified and multivariable analyses were performed to account for antiepileptic drug exposure, disease duration, and anthropometric factors, residual confounding cannot be fully excluded given the complexity of endocrine–metabolic regulation in epilepsy. In addition, although controls were comparable for age and sex and BMI-related variables were included in the analyses, more precise matching for anthropometric characteristics could have strengthened the study, and some residual confounding related to body composition cannot be excluded. In addition, treatment heterogeneity, especially the high prevalence of polytherapy, represents a further limitation. The limited size of several treatment subgroups and the overlap among different ASM combinations did not allow robust stratification according to specific ASM classes, including enzyme-inducing, non-enzyme–inducing, or valproate-containing regimens. In addition, detailed data on lifestyle-related factors, such as diet and physical activity, were not available and may have influenced metabolic outcomes. In particular, diet may affect energy balance and metabolic regulation, which in turn can modify peripheral thyroid hormone metabolism and the FT3/FT4 ratio [39, 49, 50]. Similarly, physical activity may influence energy expenditure and metabolic adaptation, potentially contributing to variability in thyroid hormone parameters [39, 51, 52]. Moreover, patients were not stratified by epilepsy syndrome or seizure type, because the study mainly focused on treatment-related effects and detailed clinical data were not available for the entire cohort. Since thyroid–metabolic interactions may differ across epilepsy syndromes, this may limit the generalizability of the findings. Larger future studies with more detailed syndrome- and seizure type–specific characterization are needed, although the present study still provides a meaningful contribution to understanding thyroid–metabolic interactions in pediatric epilepsy.

## Conclusions

Children and adolescents with epilepsy show altered peripheral thyroid hormone metabolism, marked by an increased FT3/FT4 ratio despite preserved HPT-axis regulation. This change occurs alongside an unfavorable metabolic profile and may reflect an adaptive response to epilepsy-related factors and/or long-term antiepileptic treatment. Multivariable analyses identified BMI z-score as a major independent determinant, while antiepileptic drug exposure showed variable effects, supporting a multifactorial thyroid–metabolic remodeling in pediatric epilepsy.

## Supplementary Information

Below is the link to the electronic supplementary material.

ESM 1 Supplementary Material 1 (DOCX 902 KB)

## Data Availability

All data supporting the findings of this study are available within the paper and its Supplementary Information.
