# Supplementary material for: Thyroid-metabolic interactions in pediatric epilepsy: insights from central sensitivity indices and peripheral hormone markers
Source: Eur J Pediatr. 2026 May 8;185(6):375. doi: 10.1007/s00431-026-07045-8 (PMC13156189; doi:10.1007/s00431-026-07045-8)
Supplement: Supplementary file 1 — Supplementary Material 1 (DOCX 902 KB) [file 431_2026_7045_MOESM1_ESM.docx]

**Supplementary materials**

**Supplementary material Figure 1.** Violin plots showing differences in thyroid-related parameters between epilepsy patients and healthy controls.


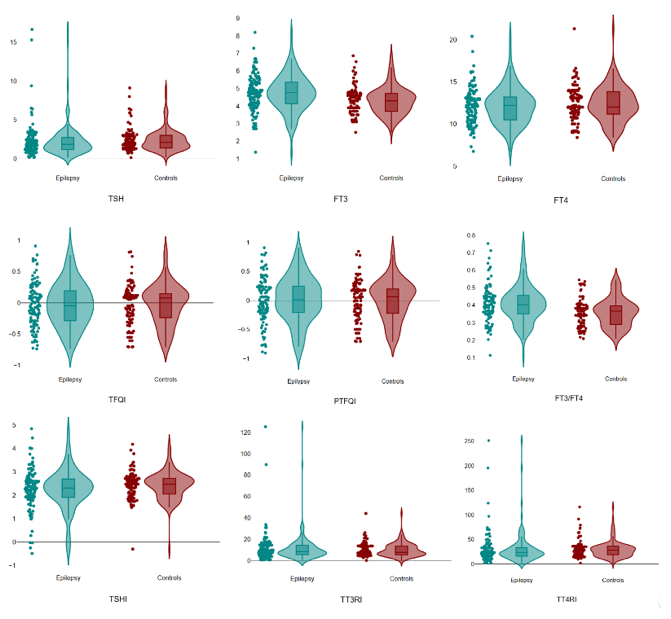


**Supplementary materials Figure 2.** Correlation matrices of the analyzed variables in the epilepsy, control, and total population groups.

**
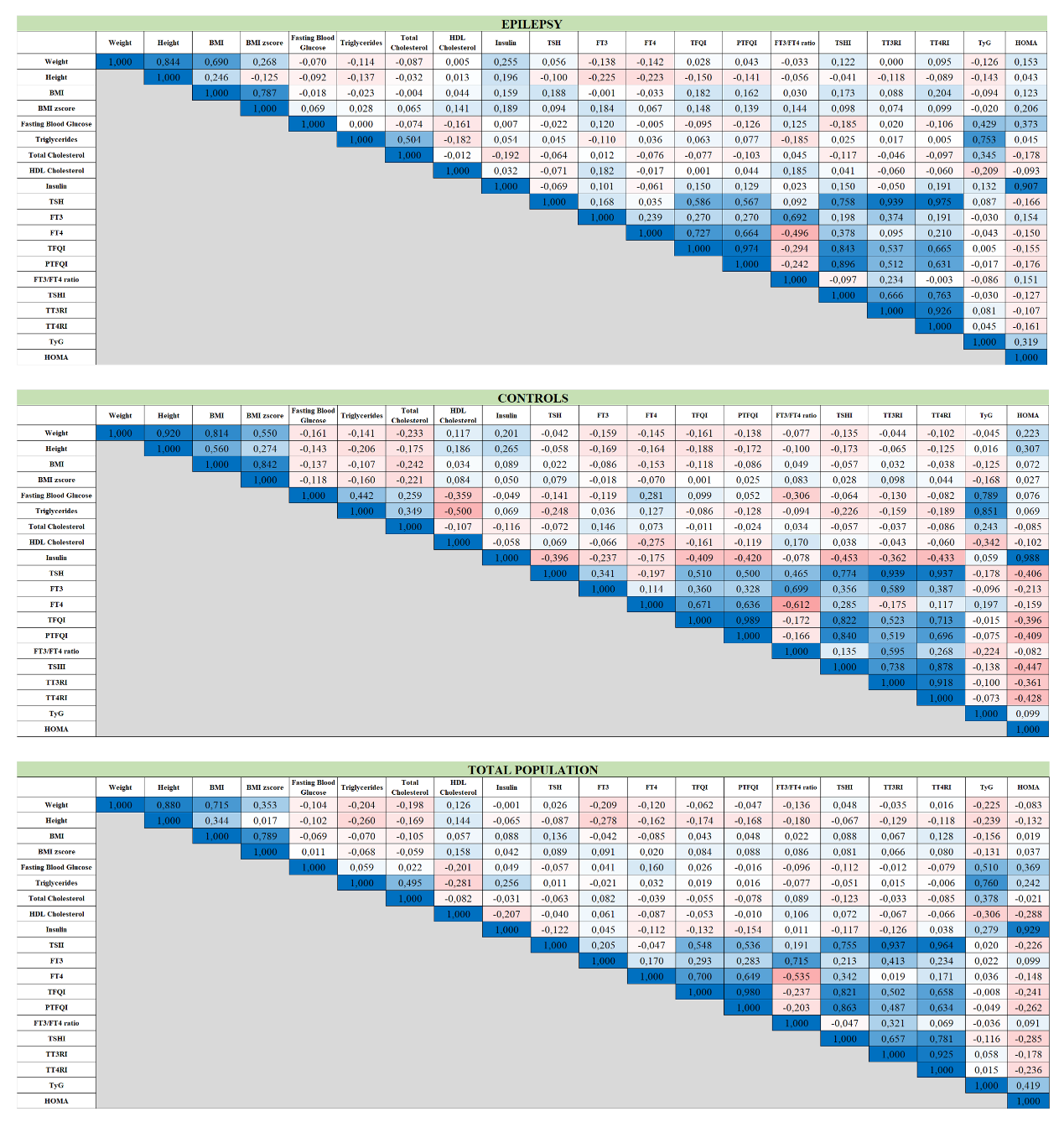
**

**Supplementary materials Figure 3.** Multivariate regression results


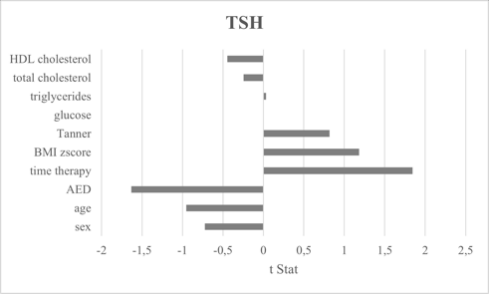

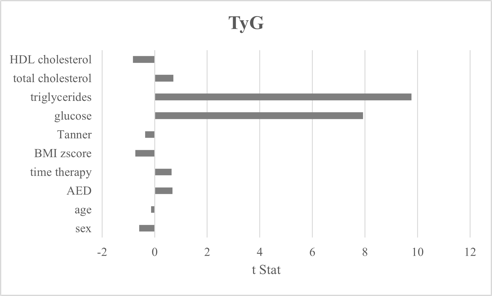

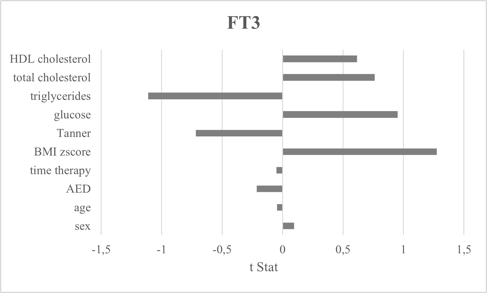


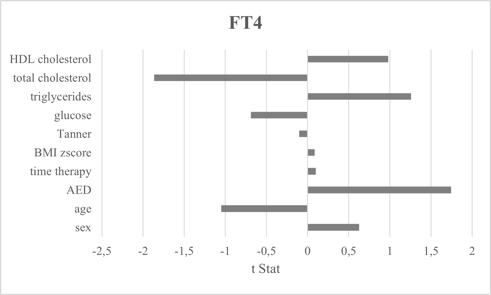

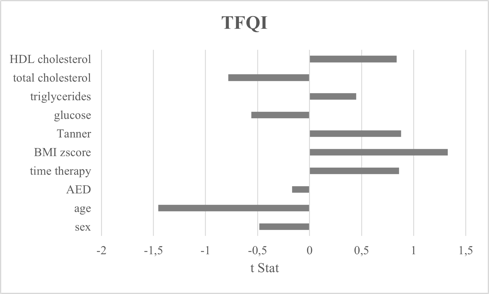

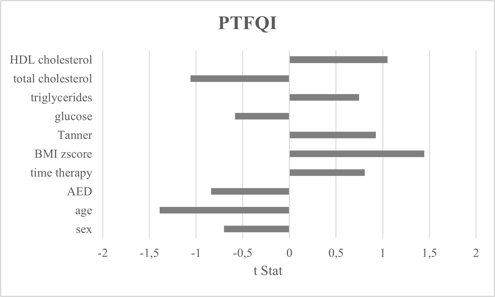


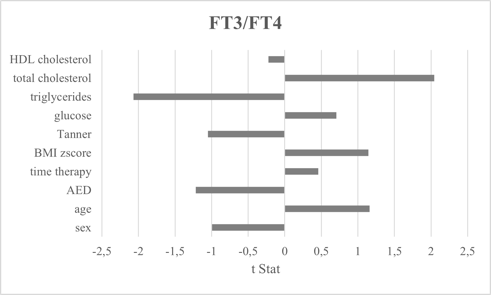

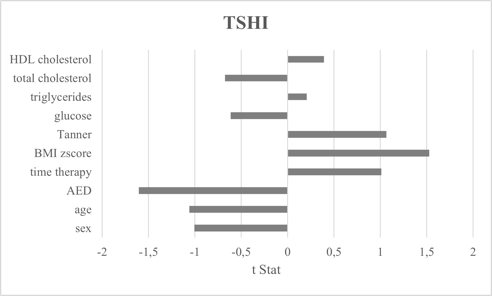

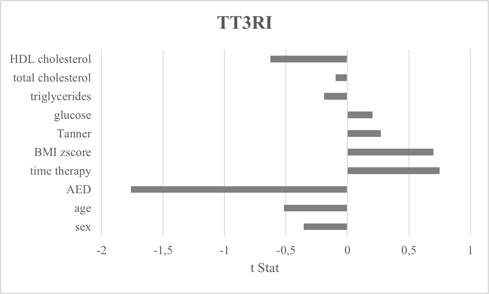


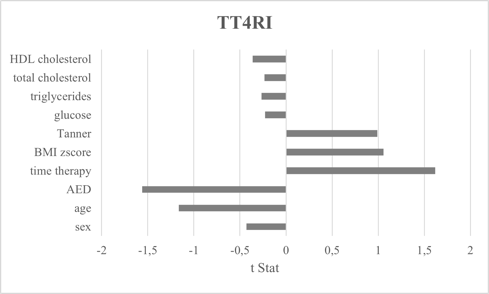

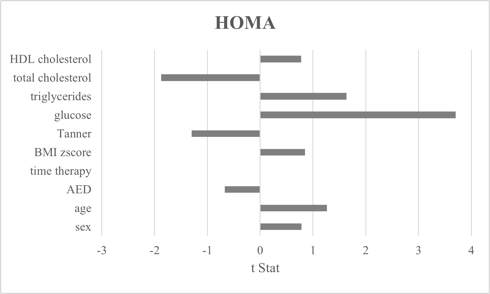


TSH = thyroid-stimulating hormone; TyG = triglyceride–glucose index; HOMA-IR = homeostasis model assessment of insulin resistance; FT3 = free triiodothyronine; FT4 = free thyroxine; TFQI = thyroid feedback quantile-based index; PTFQI = parametric thyroid feedback quantile-based index; FT3/FT4 = free triiodothyronine to free thyroxine ratio; TSHI = thyroid-stimulating hormone index; TT3RI = thyrotroph triiodothyronine resistance index; TT4RI = thyrotroph thyroxine resistance index.
